# Analysis of factors affecting pharmacists' ability to identify and solve problems

**DOI:** 10.1186/s40780-023-00300-2

**Published:** 2023-10-02

**Authors:** Keigo Watanabe, Takamasa Sakai, Fumiko Ohtsu

**Affiliations:** Drug Informatics, Faculty of Pharmacy, Meijo University, 150 Yagotoyama, Tempaku-Ku, Nagoya, Aichi Japan

**Keywords:** Pharmacists, Self-improvement, Japan, Academic conferences, Problem-solving

## Abstract

**Background:**

Among Japanese pharmacists, there is a gap in their commitment to self-improvement and a possible gap in their ability to identify and solve problems. However, the factors causing this situation have not yet been clarified. This study was conducted to identify factors that influence the abilities of Japanese pharmacists to identify and solve problems, which are skills considered essential for this profession. A prior history of presenting at academic conferences was set as a surrogate outcome to clarify whether having this experience affects the factors.

**Methods:**

A nationwide internet-based survey was conducted among 300 participating hospitals and 300 community pharmacists. The survey was discontinued when the sample size of each group reached 300. The respondents were categorized into two groups on the basis of their experience of presenting at academic conferences in the survey item “status of self-improvement after employment.” Their association with other survey items was determined using univariate and multivariate logistic regression analyses.

**Results:**

The multivariate analysis revealed that 152 (50.7%) hospital pharmacists and 41 (13.7%) community pharmacists had presented at academic conferences. Among the hospital pharmacists, the experience of presenting at academic conferences was significantly associated with the “age 30 s (*references 20 s),” “presence of pharmacists to consult,” “experience supervising interns,” and “number of types of self-improvement” factors. For the community pharmacists, prior conference presentation experience was significantly associated with “age over 70 s,” “highest educational background (doctoral or master’s degree),” and “number of types of self-improvement.”

**Conclusion:**

This pioneering study suggests that having prior experience of presenting at academic conferences as a surrogate outcome of pharmacists' problem-finding and problem-solving skills may be related to the support provided by human environmental factors at the facility, the status of self-improvement, and the highest educational background.

## Background

In recent years, the requirements of the Japanese society for pharmacists have changed significantly. In October 2015, the Ministry of Health, Labour and Welfare formulated the “Pharmacy Vision for Patients,” which requires community pharmacists to work with related institutions to provide support for anticancer drugs (advanced pharmacy management function), centralized and continuous access to medication guidance information, 24-h support, and home support [1]. Additionally, the April 2022 medical fee revision introduced refill prescriptions. For successful patient care within the refill system, a pharmacist needs to assess the patient's condition, medication adherence, the appropriateness of current medication use before dispensing medications. In addition, the pharmacist must determine if the patient requires a doctor's visit for further evaluation, and if needed, share the patient's information with the prescribing physician [2].

To be a pharmacist who can respond to these changing needs, it is essential for pharmacists to possess the skills to identify patients' medication-related problems and problem-solving skills. Those skills must be developed and demonstrated by pharmacists throughout their careers, and therefore, it is important to continue self-improvement even after employment.

Although there are various self-improvement methods, participation in workshops and e-learning is currently mainstream. However, as of December 2021, approximately 40% of pharmacists in all prefectures in Japan have received certification from the Japan Pharmacists Education Center for continuous self-improvement [3]. Writing manuscripts and presenting research findings about pharmacy practice at conferences are also part of the self-improvement process. Numerous outcome studies and papers on the effectiveness of pharmacist interventions in treatment have been published overseas, with meta-analyses reporting outcomes from interventions for blood pressure and glycemic control for patients with type 2 diabetes [4, 5]. However, a survey of hospital pharmacists at the National Hospital Organization Kyushu Group and the National Hansen's Disease Sanatorium in Japan reported that only 15.4% of pharmacists had compiled research papers. Furthermore, it has been reported that 51.8% of pharmacists have no experience in presenting at academic conferences, and only 21.4% of pharmacists are currently conducting clinical research [6]. Thus, among Japanese pharmacists, there is a gap in their commitment to self-improvement and a possible gap in their ability to identify and solve problems. This is a major problem, considering that in the future, pharmacists in Japan are required to exercise their professional abilities more comprehensively. However, the factors causing this situation have not yet been clarified.

Hence, post employment experience in presenting at academic conferences indicates proficiency in identifying, solving, and summarizing problems through research activities, In this study, experience of presenting at academic conferences, one of the self-improvement activities for pharmacists, was set as a surrogate outcome of ability to identify and solve problems, and the purpose of the study was to clarify the differences in factors depending on whether the pharmacists had this experience or not.

## Methods

### Sample size determination

The survey covered hospital pharmacists and community pharmacists nationwide. Considering that the number of survey targets for this study in 2018 [7] was approximately 60,000 hospital pharmacists and 180,000 community pharmacists, a sample size of approximately 271.02 for hospital pharmacists and 271.84 for community pharmacists was calculated assuming a 5% margin of error, 90% confidence level, and 50% response rate. Therefore, we set the required sample size to 300 hospital pharmacists and 300 community pharmacists, respectively. The survey was terminated after the initial collection of 300 responses.

### Survey method

Surveys targeting specific groups of people (i.e., pharmacists), in a specific region, whose educational and environments, motivation, etc. were similar, were at risk of response bias. Therefore, to improve external validity, a nationwide Internet survey was conducted using Loom Inc. Ltd., a company commissioned to randomly select survey targets from among registered pharmacists nationwide, distribute the survey questionnaire, obtain consent from survey participants, and collect responses to the questionnaire until the number of targets was reached.

### Survey period

The survey period was from February 7, 2022, to March 7, 2022.

### Survey items

The survey items were set to clarify the factors related to hospital and community pharmacists’ “ability to identify and solve problems.” Details, including the abbreviations of survey items, are presented in Table 1.

**Table 1:** Survey items

Survey items	
Respondent attributes	Sex [sex (male)], Age[Age: 20 s/30 s/40 s/50 s/60 s/over 70 s], Region of residence, Undergraduate courses (6-year course /4-year course), University of origin (national/private), Last educational background (bachelor's degree / doctoral or master's degree) [ Highest educational background (doctoral or master's degree)], Graduate school (national/private), Current position: Management, etc., Experience of changing jobs
Life events experienced	Marriage, Childbirth, Maternity Leave, Childcare, Nursing care, Job change, Transfer, etc
Experiences gained in pharmacy school	Experience in attending academic conferences [Experience participating in academic conferences during pharmacy school], Experience presenting at academic conferences [Experience presenting at academic conferences during pharmacy school], Paper submission experience, Experience with courses in health economics, Pharmacoeconomics, Pharmacoinformatics, pharmaco-therapeutics, etc. Pharmacoeconomics, Pharmacoeconomics, Pharmacoinformatics, Pharmacotherapeutics, etc. [Course experience during pharmacy school], PBL learning experience, Experience with laboratory assignments [Experience in laboratory assignments], Experience in writing a thesis, Experience meeting role models during clinical practice (practical training, etc.) while enrolled in pharmacy school [Meeting a role model]
Physical environmental factors at your facility	Number of full-time pharmacists at your facility: Hospital pharmacist [More than 16 full-time pharmacists], Community pharmacist [More than 3 full-time pharmacists], Facility size: Hospital pharmacist [Affiliated with large hospitals], Community pharmacist [Affiliated with pharmacy chains or DS], Frequency of information exchange with prescribing physician: Hospital pharmacist [Frequency of information exchange is more than once/week], Community pharmacist [Frequency of information exchange is more than once/month], Subsidy system for self-improvement [Subsidy system], Opportunities to report on what you have learned and experienced [Opportunities to report learning]
Human environmental factors at your facility	Presence of a pharmacist who can be consulted regarding daily operations and drug questions, A presence that can be consulted when conducting research activities such as academic conference presentations [Presence of a pharmacist to consult], Presence of colleagues who can attend academic conferences and training sessions together [Friends to join together], Experience participating in practical training workshops [Experience in participating in workshops], Experience in teaching interns in the past year [Experience in supervising interns]
The status of self-learning/ self-improvement motivation after employment	Experience presenting at academic conferences [Experience presenting at academic conferences], Number of types of self-improvement experienced in the past year (e.g., participation in workshops) [Number of types of self-improvement], Self-improvement currently being undertaken or not [Execution of self-improvement], Assignments given by your institution [Assignment from your facility], Experience participating in academic conferences, Hurdles in research activities, Willingness to present at future academic conferences, Post-employment experience submitting papers, Memberships in academic societies at this time, Experience in obtaining certifications such as certified specialty pharmacy technician advisor, Affiliation with prefectural, municipal, or other pharmacists' associations or hospital pharmacists' associations, The most important matters in engaging in research activities, lifelong learning, and other self-improvement activities

### Analysis method

Experience in presenting at academic conferences on the status of self-improvement activities after pharmacy school graduation was set as a surrogate outcome of “problem identification and solution ability” among the survey items. The survey items were categorized into two groups: those who responded that they had presented at academic conferences (group with experience presenting at academic conferences) and those who responded that they had not presented at academic conferences (group without experience presenting at academic conferences). The χ2 test for nominal data comparisons such as gender and region of residence, and the Mann–Whitney U test for numerical data comparisons such as the number of self-improvement experiences and number of life events experienced by hospital pharmacists and community pharmacists, respectively, and each survey item. Multivariate logistic regression analysis was conducted after considering multicollinearity for items with *P* < 0.2.

To examine the influence of facility affiliation, a stratified analysis was conducted by categorizing hospital pharmacists into hospitals with specific functions and community health care support (hereafter referred to as “large hospitals”) and hospitals other than those with specific functions and community health care support (hereafter referred to as “small and medium-sized hospitals”); community pharmacists were categorized into community pharmacy chains and drugstores (hereafter referred to as “pharmacy chains and DS”) and individual and small community pharmacies (hereafter referred to as “small and medium-sized community pharmacies”).

Additionally, multivariate logistic regression analysis was conducted with the item and the number of life events experienced in place of the item of sex as a sensitivity analysis. IBM SPSS Statistics Version 27 (IBM Corporation, Armonk, NY, USA) was used for analysis.

### Ethical considerations

This study has been approved by the Ethical Review Board, Faculty of Pharmacy, Meijo University (Approval No. R3-5).

## Results

### Background of respondents

The backgrounds of the hospital and community pharmacists respondents are shown in Table 2.

**Table 2:** Hospital and community pharmacists background

	Hospital pharmacist			Community pharmacist		
	Group with experience presenting at academic conferences (*N* = 152)	Group without experience presenting at academic conferences (*N* = 148)	*P* value	Group with experience presenting at academic conferences (*N* = 41)	Group without experience presenting at academic conferences (*N* = 259)	*P* value
Sex (male/female)^b^	81/71	50/98	< 0.001^a^	21/20	92/167	0.054
Age [Age 20 s/30 s/40 s/50 s/60 s/age over 70 s]^b^	15/75/32/25/5/0	32/60/28/22/6/0	0.15	2/18/12/4/3/2	34/93/74/44/13/1	0.54
Region of residence (Hokkaido region /Tohoku region/ Kanto region / Chubu region / Kinki region / Chugoku region / Shikoku region / Kyushu region)^b^	9/5/ 43/35/25/ 8/2/25	9/3/ 49/29/26/ 4/5/23	0.77	3/0/ 12/9/9/ 2/0/6	6/6/ 102/37/47/ 21/9/31	0.28
Undergraduate courses (6-year course /4-year course)^b^	65/87	73/75	0.25	14/27	91/168	0.90
Highest educational background (bachelor's/doctoral or master’s degree)^b^	118/34	136/12	< 0.001^a^	30/11	237/22	< 0.001^a^
Facility size (Hospital pharmacist: large hospital/ small- and medium-sized hospitals, Community pharmacist: pharmacy chains or DS/small- and medium-sized community pharmacies)^b^	85/67	40/108	< 0.001^a^	22/19	132/127	0.75
Current position: Management, etc. (with/without position)^b^	60/92	42/106	0.04^a^	19/22	102/157	0.40
Life events experienced (quartile range)^c^	2(1–4)	2(1–4)	0.49	3(1–4)	3(1–5)	0.41

^a^Significant at *P* < 0.05

^b^χ2 test

^c^Mann–Whitney U test

The “group with experience presenting at academic conferences” consisted of 152 hospital pharmacists (50.7%) and the “group without experience presenting at academic conferences” consisted of 148 hospital pharmacists (49.3%). The survey items that showed differences in respondents’ backgrounds between the “group with experience presenting at academic conferences” and the “group without experience presenting at academic conferences” were sex (*P* < 0.001) etc.

Next, the “group with experience presenting at academic conferences” consisted of 41 community pharmacists (13.7%) and the “group without experience presenting at academic conferences” consisted of 259 community pharmacists (86.3%). The only difference in the backgrounds between these two groups was in their highest educational background(bachelor's/master’s or doctoral degree) (*P* < 0.001).

### Multivariate logistic regression analysis of hospital and community pharmacists

The results of the multivariate logistic regression analysis of hospital pharmacists and community pharmacists are presented in Table 3. Among hospital pharmacists, the survey items that showed significant positive associations with “group with experience presenting at academic conferences” were “presence of pharmacists to consult” etc.

**Table 3:** Results of multivariate logistic regression analysis in hospital and community pharmacists

Hospital pharmacist	Group with experience presenting at academic conferences (*N* = 152)		Group without experience presenting at academic conferences (*N* = 148)				
	N	%	N	%	OR	95%CI	*P* value
Respondent attributes							
Sex (male)	81	53.3	50	33.8	1.9	1.0–3.7	0.06
Age 20 s	15	9.9	32	21.6	Ref	-	-
30 s	75	49.3	60	40.5	2.8	1.2–6.6	0.02^a^
40 s	32	21.1	28	18.9	2.5	0.8–7.9	0.12
50 s	25	16.4	22	14.9	2.4	0.7–8.7	0.17
60 s	5	3.3	6	4.1	2.1	0.4–11.0	0.40
Highest educational background (doctoral or master's degree)	34	22.4	12	8.1	1.9	0.8–4.6	0.17
Experiences gained in pharmacy school							
Experience presenting at academic conferences during pharmacy school	52	34.2	30	20.3	1.1	0.5–2.1	0.86
Course experience while enrolled in pharmacy school (quartile range)	4(2–6)		4(1–6)		1.0	0.9–1.1	0.51
Experience in laboratory assignments	151	99.3	144	97.3	3.7	0.2–57.5	0.35
Meeting a role model	88	57.9	103	69.6	1.8	1.0–3.5	0.70
Physical environmental factors at your facility							
Affiliated with large hospitals	85	55.9	40	27.1	1.5	0.8–2.7	0.24
Frequency of information exchange is more than once/week	117	77.0	95	64.2	1.1	0.6–2.0	0.83
Subsidy system	81	53.3	56	37.8	1.0	0.6–1.8	0.99
Opportunities to report learning	95	62.5	51	34.5	1.6	0.9–3.0	0.11
Human environmental factors at your facility							
Presence of a pharmacist to consult	119	78.3	61	41.2	2.4	1.2–4.6	0.01^a^
Experience in supervising interns	114	75.0	55	37.2	2.5	1.3–4.7	0.003^a^
The status of self-learning /self-improvement motivation after employment							
Number of types of self-improvement (quartile range)	4(2–5.75)		2(1–3)		1.3	1.1–1.6	0.008^a^
Execution of self-improvement	104	68.4	51	34.5	1.0	0.5–2.0	0.96
Community pharmacist	Group with experience presenting at academic conferences (*N* = 41)		Group without experience presenting at academic conferences (*N* = 259)				
	N	%	N	%	OR	95%CI	*P* value
Respondent attributes							
Sex (male)	21	51.2	92	35.5	1.6	0.7–3.3	0.25
Age 20 s	2	4.9	34	13.1	Ref	-	-
30 s	18	43.9	93	35.9	2.4	0.5–12.1	0.29
40 s	12	29.3	74	28.6	2.4	0.4–13.8	0.33
50 s	4	9.8	44	17.0	1.5	0.2–11.4	0.68
60 s	3	7.3	13	5.0	3.5	0.4–30.7	0.26
over 70 s	2	4.9	1	0.4	23.2	1.0–522.7	0.048^a^
Highest educational background (doctoral or master's degree)	11	26.8	22	8.5	2.7	1.0–7.3	0.049^a^
Experiences gained in pharmacy school							
Experience participating in academic conferences during pharmacy school	17	41.5	63	24.3	1.8	0.7–4.2	0.20
Course experience while enrolled in pharmacy school (quartile range)	3(1.5–5)		3(1–5)		1.0	0.8–1.1	0.62
Meeting a role model	17	41.5	63	24.3	2.0	0.9–4.8	0.10
Physical environmental factors at your facility							
Affiliated with pharmacy chains or DS	22	53.7	132	51.0	1.2	0.5–2.6	0.66
Frequency of information exchange is more than once/month	28	68.3	140	54.1	1.0	0.4–2.2	0.10
Subsidy system	30	73.2	155	59.8	1.9	0.8–4.4	0.15
Opportunities to report learning	15	36.6	35	13.5	2.1	0.9–5.1	0.10
Human environmental factors at your facility							
Presence of a pharmacist to consult	21	51.2	81	31.3	0.7	0.2–2.6	0.64
Experience in participating in workshops	12	29.3	50	19.3	0.9	0.4–2.3	0.85
The status of self-learning /self-improvement motivation after employment							
Number of types of self-improvement (quartile range)	4(2–4.5)		2(1–3)		1.4	1.1–1.7	0.005^a^
Execution of self-improvement	21	51.2	102	39.4	0.8	0.4–1.8	0.60

*OR* odds ratio, *CI* confidence interval

^a^Significant at *P* < 0.05

Among community pharmacists, the survey items that showed significant positive associations with “group with experience presenting at academic conferences” were “number of types of self-improvement” etc.

### Stratified analysis by facility size

Table 4 shows the results of the multivariate logistic regression analysis of pharmacists affiliated with large or small- and medium-sized hospitals. The survey items that showed significant positive associations with the “group with experience presenting at academic conferences” among pharmacists affiliated with large hospitals were “sex (male)” etc.

**Table 4:** Pharmacists affiliated with large or small- and medium-sized hospitals (Stratified analysis)

	Affiliated with large hospitals							Affiliated with small- and medium-sized hospitals						
	Group with experience presenting at academic conferences (*N* = 85)		Group without experience presenting at academic conferences (*N* = 40)					Group with experience presenting at academic conferences (*N* = 67)		Group without experience presenting at academic conferences (*N* = 108)				
	N	%	N	%	OR	95%CI	*P* value	N	%	N	%	OR	95%CI	*P* value
Respondent attributes														
Sex (male)	51	60.0	12	30.0	4.8	1.5–15.4	0.008^a^	30	44.8	38	35.2	1.4	0.5–3.9	0.49
Age 20 s	10	11.8	12	30.0	Ref	-	-	5	7.5	20	18.5	Ref	-	-
30 s	46	54.1	19	47.5	1.8	0.5–6.7	0.38	29	43.3	41	38.0	4.4	1.1–16.9	0.03^a^
40 s	15	17.6	5	12.5	0.7	0.1–4.7	0.75	17	25.4	23	21.3	6.2	1.3–28.9	0.02^a^
50 s	13	15.3	3	7.5	1.1	0.1–8.7	0.96	12	17.9	19	17.6	9.3	1.6–52.5	0.01^a^
60 s	1	1.2	1	2.5	0.6	0–16.5	0.75	4	6.0	5	4.6	6.5	0.7–57.4	0.09
Highest educational background (doctoral or master's degree)	21	24.7	4	10.0	2.3	0.5–10.2	0.28	13	19.4	8	7.4	2.2	0.6–7.8	0.24
Experiences gained in pharmacy school														
Experience presenting at academic conferences during pharmacy school	34	40.0	13	32.5	1.0	0.3–3.1	0.95	18	26.9	17	15.7	1.3	0.4–3.7	0.66
Meeting a role model	35	41.2	17	42.5	1.5	0.5–4.1	0.47	29	43.3	28	25.9	3.2	1.2–8.3	0.02^a^
Physical environmental factors at your facility														
More than 16 full-time pharmacists	72	84.7	31	77.5	1.0	0.3–4.0	0.96	31	46.3	23	21.3	3.5	1.2–10.4	0.02^a^
Frequency of information exchange is more than once/week	63	74.1	29	72.5	0.6	0.2–1.8	0.34	54	80.6	66	61.1	2.1	0.9–5.2	0.10
Subsidy system	48	56.5	21	52.5	0.6	0.2–1.7	0.36	33	49.3	35	32.4	1.6	0.7–3.5	0.28
Opportunities to report learning	57	67.1	19	47.5	1.9	0.6–6.1	0.25	38	56.7	32	29.6	1.4	0.6–3.2	0.41
Human environmental factors at your facility														
Presence of a pharmacist to consult	71	83.5	31	77.5	1.3	0.4–4.6	0.70	48	71.6	30	27.8	2.5	1.0–6.0	0.04^a^
Experience in supervising interns	72	84.7	24	60.0	1.9	0.6–5.8	0.28	42	62.7	31	28.7	1.7	0.7–4.5	0.24
The status of self-learning /self-improvement motivation after employment														
Number of types of self-improvement (quartile range)	4(3–6)		3(2–4)		1.6	1.2–2.2	0.002^a^	3(2–4)		2(1–3)		1.2	0.9–1.6	0.26
Execution of self-improvement	64	75.3	23	57.5	0.7	0.2–2.1	0.52	40	59.7	28	25.9	1.2	0.4–3.2	0.75
Assignment from your facility	37	43.5	14	35.0	1.0	0.4–2.9	0.97	25	37.3	23	21.3	1.1	0.5–2.8	0.78

*OR* odds ratio, *CI* confidence interval

^a^Significant at *P* < 0.05

The survey items that showed significant positive associations with the “group with experience presenting at academic conferences” among pharmacists belonging to small- and medium-sized hospitals were “presence of a pharmacist to consult” etc.

The results of the multivariate logistic regression analysis for pharmacists affiliated with pharmacy chains and DS or small- and medium-sized community pharmacies are shown in Table 5. The survey item that showed a significant positive association with the “group with experience presenting at academic conferences” among pharmacists affiliated with pharmacy chains and DS was the highest educational background (“doctoral or master's degree”).

**Table 5:** Pharmacists affiliated with pharmacy chains/DS or small- and medium-sized community pharmacies (Stratified analysis)

	Affiliated with pharmacy chains or DS							Affiliated with small- and medium-sized community pharmacies						
	Group with experience presenting at academic conferences (*N* = 22)		Group without experience presenting at academic conferences (*N* = 132)					Group with experience presenting at academic conferences (*N* = 19)		Group without experience presenting at academic conferences (*N* = 127)				
	N	%	N	%	OR	95%CI	*P* value	N	%	N	%	OR	95%CI	*P* value
Respondent attributes														
Sex (male)	11	50.0	41	31.1	1.6	0.5–4.9	0.45	10	52.6	51	40.2	2.0	0.5–7.4	0.30
Age 20 s	2	9.1	27	20.5	Ref	-	-	0	0	7	5.5	0	0	> 0.99
30 s	10	45.5	48	36.4	1.4	0.2–8.3	0.69	8	42.1	45	35.4	Ref	-	-
40 s	6	27.3	35	26.5	1.5	0.2–10.8	0.69	6	31.6	39	30.7	1.4	0.3–5.8	0.67
50 s	2	9.1	17	12.9	0.7	0.1–7.9	0.76	2	10.5	27	21.3	0.4	0.1–3.0	0.38
60 s	1	4.5	4	3.0	1.0	0–21.7	0.99	2	10.5	9	7.1	4.2	0.5–36.7	0.19
over 70 s	1	4.5	1	0.8	2.3	0.1–97.1	0.67	1	5.3	0	0	8.7 × 10^10^	0	> 0.99
Highest educational background (doctoral or master's degree)	6	27.3	10	7.6	8.0	1.4–46.4	0.02^a^	5	26.3	12	9.4	1.4	0.3–6.6	0.71
Experiences gained in pharmacy school														
Experience participating in academic conferences during pharmacy school	10	45.5	36	27.3	1.1	0.3–3.9	0.88	7	36.8	27	21.3	2.3	0.6–9.1	0.24
Meeting a role model	8	36.4	32	24.2	1.5	0.4–5.6	0.57	9	47.4	31	22.4	3.2	0.7–14.6	0.14
Physical environmental factors at your facility														
More than 3 full-time pharmacists	11	50.0	39	29.5	0.4	0.1–1.2	0.09	12	63.2	64	50.4	1.3	0.3–5.0	0.70
Frequency of information exchange is more than once/month	14	63.6	68	51.5	1.3	0.4–4.2	0.65	14	73.7	72	56.7	0.7	0.2–2.8	0.63
Subsidy system	17	77.3	94	71.2	1.9	0.5–7.1	0.35	13	68.4	61	48.0	3.2	0.7–14.6	0.14
Opportunities to report learning	7	31.8	19	14.4	1.3	0.3–5.2	0.73	8	42.1	16	12.6	4.1	0.9–19.3	0.07
Human environmental factors at your facility														
Friends to join together	10	45.5	41	31.1	2.1	0.6–7.7	0.26	11	57.9	38	29.9	1.0	0.2–4.5	0.96
Experience in participating in workshops	5	22.7	27	20.5	0.6	0.1–2.6	0.49	7	36.8	23	18.1	0.8	0.2–3.9	0.81
The status of self-learning /self-improvement motivatio after employment														
Number of types of self-improvement (quartile range)	3(2–4)		2(1–3)		1.4	1.0–1.9	0.09	4(2–5)		2(1–4)		1.5	1.0–2.1	0.04^a^
Execution of self-improvement	9	40.9	59	44.7	0.4	0.1–1.2	0.09	12	63.2	43	33.9	1.8	0.5–6.9	0.37

*OR* odds ratio, *CI* confidence interval

^a^Significant at *P* < 0.05

The survey item that showed a significant positive association with the “group with experience presenting at academic conferences” among pharmacists affiliated with small- and medium-sized community pharmacies was “number of types of self-improvement”.

### Sensitivity analysis

As a sensitivity analysis, for each of the hospital pharmacists and community pharmacists, the item “sex” was excluded from this analysis, and instead the item “number of life events experienced” was used in the multivariate logistic regression analysis. For hospital pharmacists, the survey items that recently showed a significant positive association with the “group with experience presenting at academic conferences” by sensitivity analysis were “age 40 s (*references 20 s)” and “age 50 s” of the respondent background. For community pharmacists, the sensitivity analysis revealed that no new survey item showed a significant positive association with the “group with experience presenting at academic conferences,” but the highest educational background (“doctoral or master 's degree”), which had previously been considered associated, was no longer considered so.

## Discussion

There was no significant bias in respondents' backgrounds in terms of age, undergraduate course, or region of residence. However, differences were found in terms of sex and highest educational background.

The results of multivariate logistic regression analysis of hospital pharmacists revealed significant associations between “experience presenting at academic conferences” and “age 30 s,” “presence of pharmacists to consult,” “experience in supervising interns,” and “number of types of self-improvement.”

In a report on clinical research by hospital pharmacists, 94.6% of the participants indicated that they were anxious when planning and conducting clinical research. They cited “statistical analysis,” “preparation of research protocol,” and “how to formulate clinical questions” as points of anxiety (or uncertainty) [6]. Therefore, the results of this study highlight the importance of having a pharmacist available at your facility for research consultations, enabling the presentation of your research findings at academic conferences. Additionally, according to the Learning Pyramid published by the US National Institute for Training and Research, teaching others is a learning method with the highest knowledge retention rate [8]. Muroi reported that all pharmacists in a hospital have developed a roof-tile educational system in which they can learn and grow together with interns, thereby deepening their own understanding of diseases and drug therapy [9]. Therefore, we believe that the experience of supervising interns stimulates motivation and reflection on one's own knowledge, which may be related to academic conference presentations. Among hospital pharmacists, support from the human environment of the facility and motivation for self-improvement may be important factors in their commitment to presenting at academic conferences.

However, the items that showed significant associations with the results for community pharmacists were “over 70 s,” “highest educational background (doctoral or master's degree),” and “number of types of self-improvement.” No significant associations were found between physical or human environmental factors in the facility to which they belonged. Thus, among community pharmacists, physical and human environmental factors did not differ significantly among pharmacies, suggesting that individual motivation influenced their commitment to present at academic conferences. In a survey of Canadian pharmacists working in emergency medicine, there was a relationship between current research and the experience of obtaining a master's or doctoral degree [10], and it is thought that their original motivation for research, such as the experience of attending a master's or doctoral program, influenced their commitment to presenting at academic conferences after employment. However, the small number of community pharmacists with experience presenting at academic conferences suggests that this factor may not have been accurately captured.

Regarding the results of the stratified analysis, significant associations were found for “sex (male)” and the “number of types of self-improvement” among pharmacists affiliated with large hospitals. Ueki et al. reported that the greater the number of hospital beds, the lower the levels of job satisfaction [11]. Women are less satisfied with their jobs than men are in items such as relationships with other healthcare professionals and patients. Furthermore, they may take leaves of absence or resign from their jobs owing to life events such as childbirth or maternity leave. Therefore, it is possible that men work more persistently at large hospitals and, consequently, have more opportunities to present at academic conferences. Additionally, since environmental factors, such as guidance and education systems, are generally considered better in large hospitals, it is possible that individual motivation for self-improvement influences one’s efforts to present at academic conferences. Significant associations were found among pharmacists belonging to “ 30 s,” “40 s,” “50 s,” “meeting a role model,” “more than 16 full-time pharmacists,” and “presence of a pharmacist to consult” among pharmacists belonging to small- and medium-sized hospitals. However, no significant association was found for “number of types of self-improvement.” Therefore, it was considered that in small- and medium-sized hospitals, the physical and human environmental factors of their institutions, such as stimulating experiences as students, consultation systems, and personnel, influenced pharmacists’ commitment to presenting at academic conferences rather than their motivation to conduct self-improvement.

A survey on clinical research support efforts reported that the number of oral presentations at academic conferences and the number of papers written by hospital pharmacists improved when universities and civil hospitals collaborated and university faculties provided research guidance to hospital pharmacists [12]. Since small- and medium-sized hospitals do not have the same physical and human environment as large hospitals, it is conceivable that the establishment of such support may increase opportunities to make presentations at academic conferences.

Accordingly, pharmacists in small- and medium-sized hospitals may be constrained from taking part in conference presentation efforts because of a lack of a specific number of years of experience or a sufficient number of pharmacists. It is possible that those in their 20 s may not have had many opportunities to experience academic conference presentations, as the focus of their work is on daily duties. However, the “presence of a pharmacist to consult” and “meeting a role model” may have positive impacts.

It was interesting that “meeting a role model” was related to the experience of presenting at academic conferences in an environment with insufficient physical and human resources at one's facility.

In the stratified analysis of community pharmacists, only the highest educational background (“doctoral or master 's degree”) was significantly associated with pharmacists affiliated with a pharmacy chains or DS. Regarding motivation for self-improvement, it is possible that pharmacists affiliated with pharmacy chains and DS have a better environment for self-improvement, such as training, than small- and medium-sized community pharmacies, and that no difference was found. Therefore, it is conceivable that only past research experience may have led to their commitment to academic conference presentations. However, the small number of community pharmacists who had　presented at academic conferences may not have accurately captured these factors.

A significant association was found between number of types of self-improvement among pharmacists belonging to small- and medium-sized community pharmacies. Therefore, it is possible that pharmacists belonging to small- and medium-sized community pharmacies have greater differences in motivation for self-improvement, which may affect their commitment to academic conference presentations.

Additionally, a comparison of hospital pharmacists and community pharmacists showed a significant difference in the background of the respondents, with only 41 (13.7%) of the community pharmacists having “experience presenting at academic conferences” compared to 152 (50.7%) of the hospital pharmacists. Compared to the latter, the former are expected to face multiple physical environmental and human environmental factors at your facility when presenting at academic conferences. Hospital pharmacists can obtain detailed patient information, such as background, medical history, and laboratory values from medical records. However, community pharmacists will only be able to access limited information from prescriptions and medication records.

Additionally, according to a survey [13] by the Ministry of Health, Labour and Welfare, the average number of pharmacists in one pharmacy was 2.3 (median 2.0); there may be insufficient pharmacists to guide on research owing to fewer pharmacists in the facility compared to hospitals. In the present results, there was a significant association between “experience presenting at academic conferences” and “presence of pharmacists to consult” among hospital pharmacists, but not among community pharmacists. Therefore, it is possible that the presence of mentors was low among community pharmacists. These differences in human environmental factors may be responsible for differences in academic conference presentation experiences between hospital and community pharmacists.

Sato et al. conducted a clinical study as a community pharmacists, in collaboration with hospital pharmacy departments and universities and reported the results [14, 15]. In the United States, the Governmental Agency for Healthcare Research and Quality has taken the lead in establishing practice-based studies. Since 1999, a practice-based research network has been established in each region, led by primary care physicians. Pharmacists' awareness, interest, and motivation in clinical research have reportedly increased over time, owing to the existence of such networks [16]. To address the strain in conducting research in affiliated institutions' current state of affairs, we suggest forming networks and collaborating with other facilities and institutions, as in the case of small- and medium-sized hospitals. We believe that this strategy can be adopted in Japan.

In the sensitivity analysis, there was no significant variation in items other than respondent attributes (age, last educational background, etc.) for hospital and community pharmacists. Therefore, the results of this analysis were considered robust. The percentage of hospital pharmacists who had presented at academic conferences was similar to that in previous studies [6].

Since this study was a nationwide, Internet-based survey, a limitation was the inclusion of only pharmacists registered as monitors with an Internet research firm and of those able to use a PC or other electronic devices. However, the use of electronic devices is currently mandatory in pharmacist practices. As there was no bias in respondent age, we were able to collect results that were close to the current situations of pharmacists. Based on the results of a survey of hospital pharmacists regarding the surrogate outcomes of this study [6], we considered that the absolute number of hospital and community pharmacists with experience in writing papers is currently small.

Therefore, in order to emphasize the feasibility of the study, we set the experience of presenting at academic conferences, which is considered the first step in evidence generation, as an outcome in this study, although it is a lower hurdle compared to writing a paper. Therefore, factors other than the experience of writing may exist.

In addition, we did not confirm the affiliations and number of presentations for the surrogate outcome in this study, which was the presence or absence of experience presenting at academic conferences after employment. Therefore, it is possible that employment history influenced the results or that the presentation was a one-time event. Therefore, it is unclear whether the problem was continuously identified and resolved. And we did not investigate the quality of evidence for the content of past conference presentations.

The number of pharmacists who had presented at academic conferences was extremely small among community pharmacists, and the sample size was not large enough to accommodate the number of factors in the multivariate analysis. This limitation reduced the reliability of the analysis.

## Conclusion

In this study, the experience of presenting at academic conferences after employment was examined as an indicator of problem-solving ability.

The results revealed that factors influencing pharmacists’ experiences post-employment and presenting at academic conferences might include sex, the highest educational background, experience during pharmacy school, the human and physical environment of the institution, and the status of self-improvement. The factors differed by hospital, pharmacy, and the size of each institution. Among the factors newly identified in this study, “presence of a pharmacist to consult,” “experience in supervising interns,” and “meeting a role model” were associated with improved practical knowledge in each environment. To improve this situation, it is necessary to consider measures to motivate pharmacists for self-improvement as well as attempts to accumulate practical knowledge and share it among pharmacists in the future.

## Data Availability

All data generated or analyzed in this study are included in the manuscript.
